# Artificial Intelligence in Transcriptomics: From Human-in-the-Loop to Agentic AI

**DOI:** 10.3390/jpm16040181

**Published:** 2026-03-27

**Authors:** Giulia Gentile, Giovanna Morello, Valentina La Cognata, Maria Guarnaccia, Sebastiano Cavallaro

**Affiliations:** Institute for Biomedical Research and Innovation, National Research Council (CNR-IRIB), Via P. Gaifami, 18, 95126 Catania, Italy; giulia.gentile@cnr.it (G.G.); giovannamariaalessandra.morello@cnr.it (G.M.); valentina.lacognata@cnr.it (V.L.C.); maria.guarnaccia@cnr.it (M.G.)

**Keywords:** transcriptomics, functional genomics, personalized medicine, precision medicine, artificial intelligence, machine learning, reinforcement learning, deep learning, generative AI, agentic AI

## Abstract

To better understand the complexity of biological systems, research has shifted from a reductionist to a holistic approach, expanding the focus from single genes to a genome-scale view of gene activity and regulation. This is known as transcriptomics, a continuously growing field generating gene expression signatures from different technologies. A comparable paradigm shift has occurred in computational systems biology with the implementation of Artificial Intelligence (AI) learning models for gene expression analysis and integration. These models enable transcriptome-based profiling to address challenges of data heterogeneity, integration, and updating, assisting human intelligence and enhancing their ability to retrieve, analyze, integrate, and generate data recursively, thanks to their intrinsic predictive, inferential, reinforcement, and generative capabilities. Additionally, while scientists worldwide are still learning how to leverage AI methods that can maintain the human-in-the-loop, a new fundamental change is emerging: agentic AI, which can autonomously act and employ other AI methods to pursue its objectives. As a futuristic perspective, the proposed data analysis pipeline imagines agentic AI systems allowing the automated retrieval and pre-processing of heterogeneous transcriptomics data, analysis and integration with other omics datasets, performed with an incremental updating and recurrent analysis (IURA) model that could allow the detection of guideline updates (e.g., disease reclassification) and the generation of new hypotheses, such as candidate biomarkers or transcriptome–phenotype correlations. Since personalized medicine could derive profound benefits from its use, this scenario also raises important considerations regarding the advantages and concerns associated with the use of scientific AI agents in research and clinical practice.

## 1. Transcriptomics and Its Challenges

Transcriptomics captures the functional output of the genome by assessing gene expression activity and regulatory dynamics on a large scale. Advances in high-throughput sequencing now enable the generation of diverse RNA-sequencing datasets, including bulk tissue whole-genome profiles, as well as spatiotemporal and cell type-specific transcriptomic data. These technologies are dramatically increasing the volume of available data, which complements and expands upon the gene expression datasets generated over previous decades using earlier high-throughput platforms [1,2,3].

Ten years ago, genomics had been identified as a Big Data science and characterized by four domains: data acquisition, storage, distribution, and analysis [4]. Nowadays, the field of functional genomics has the same features, starting with the huge amount of data stored in different gene expression profiling repositories. Within them, data are produced using different technologies and are related to different species, diseases, tissues, and even cell types. Among these databases, those that are brain- [5] and tumor-related [6] are the most consistent. A curated but not exhaustive selection of public functional genomics databases is reported in Table 1, highlighting their heterogeneity in terms of data collection and production techniques. Some of these contain datasets belonging to pre-RNA sequencing (pre-RNA-seq) transcriptomic technologies [i.e., Microarray, Serial Analysis of Gene Expression (SAGE), Expressed Sequence Tag (EST), and In Situ Hybridization (ISH)] and, except for a few cases, are no longer updated. Others continue to increase their number of entries from microarray, bulk, single-cell, single-nucleus, and spatial RNA-seq. Moreover, each of the repositories classified in Table 1 has different protocols for data acquisition, storage, distribution, and analysis depending on the transcriptomic technology used, and thus, confirms transcriptomics as a Big Data science. Since the repositories shown in Table 1 are all public, they do not have access restrictions in downloading data, even if some of them limit access to the metadata due to ethical and legal restrictions on human research subjects [7]. Furthermore, databases generated within funded projects are no longer updated once the project has ended, although their data remains available for download.

**Figure 1:**
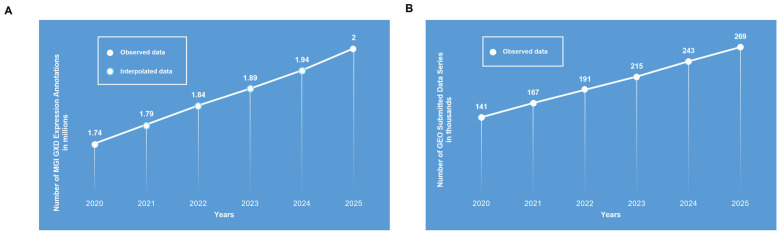
MGI GXD and GEO databases growth in the last six years. (**A**) Observational number of MGI GXD expression annotations (in millions) between 2020 and 2025, as extrapolated from [8,9]; values for years 2021–2024 were estimated by linear interpolation between observed data points due to missing annotations for those years. No formal uncertainty estimation was performed for the interpolated values; therefore, they should be considered as approximate indicators of a trend than precise measurements that cannot account for potential year-to-year variability. (**B**) Observational number of GEO submitted data series (in thousands); retrieved from https://www.ncbi.nlm.nih.gov/geo/summary/?type=history (accessed on 20 January 2026) reporting data regarding the fourth quarter of each year.

Among the public functional genomics repositories (Table 1), the most enduring are the curated resources Mouse Genome Informatics Gene Expression Database (MGI GXD) and Gene Expression Omnibus (GEO), with their initial release online in 1998 and 2000, respectively [1,8]. Over the last six years, their activity and size have grown—MGI GXD with integrated expression annotations and GEO with data series submissions—as illustrated in Figure 1. In particular, the reported growth rate of data series submitted to GEO (15% per year) reached 270k in 2025, almost doubling every five years [1]. This growth trend is due to the rapid evolution of next-generation sequencing techniques and the subsequent production of RNA-seq data. Over the years, this has changed the composition of data reported in both databases from a majority of pre-RNA-seq to a predominance of next-generation RNA-seq techniques [1,8,9], which is in accordance with reported projections of DNA-seq data growth and composition in the genomics field in the same period [4].

Transcriptomics allows basic research to be translated to the patient’s bedside, using biomarkers or pattern expression identification to stratify patients for diagnostic, prognostic, and therapeutic purposes. These results have been translated into clinical recommendations for the use of several commercially available assays for patient stratifications in different types of cancer, such as metastatic risk assessments in breast cancer as microarrays or real-time assays. In particular, those related to breast cancer have had their use recommended by the European Society for Medical Oncology in their clinical practice guidelines, receiving the highest level of evidence out of six (level I) and the highest grades of recommendation out of five (grades A and B) [10,11,12,13,14,15,16]. However, as shown above, the first challenge for researchers aiming to investigate transcriptome is represented by the overwhelming amount and heterogeneity of data produced and stored in public databases (Table 1 and Figure 1). Heterogeneity not only refers to data production techniques and the subsequently required harmonization (from different platforms) or normalization (from the same platform) of datasets (data pre-processing) [17], but also relates to issues regarding technical variations introduced during data generation, known as batch effects [18]; missing, incomplete, or incorrectly annotated metadata in databases affecting data retrieval and meta-analysis [19], or poor data quality [16,20] and imbalanced study design as variability sources non-biologically dependent. Furthermore, the incomplete automation of experimental phases introduces additional operator-dependent variability [16]; while among those biologically related, the biological starting materials used (single cells vs tissues) [21] and sex-specific transcriptional profiles represent other sources of variability [22]. In particular, transcriptomic sex-specific differences in disease development and comorbidities have been recently investigated using transcriptomic data available in public databases (with information limited in terms of availability and quality), confirming that biological sex should be considered in the design of therapeutic strategies and drug administration [23]. Comprehensively, these aspects can have an impact on data analysis, producing non-comparable data and affecting the reproducibility, validity, and biological interpretation of high-throughput gene expression studies. Non-biological and biological sources of variability in gene expression profiles are summarized in Table 2. For the aforementioned reasons, minimal requirements for data sharing of repositories, data submission of microarrays, and next-generation RNA-seq data have been implemented over the years, such as the minimum information about a microarray experiment (MIAME) and the minimal information about a high-throughput nucleotide sequencing experiment (MINSEQE) guidelines [7].

Another important challenge is represented by multi-layer omics complexity, of which transcriptomics is a single layer; it needs to be integrated and analyzed with the other omics to decode cellular states in a multi-layer network, across species, development stages, diseases, and disease stages [24]. This brings us to the second aspect of the problem: the complexity of data integration from different omics datasets. To allow the integration of multi-layer information, data need to be retrieved and pre-processed to address and remove bias deriving from the use of different platforms and experimental procedures, and then analyzed [3]. A feasible genomics integration starts from a data retrieval phase largely depending on accessibility and the update of information collected in databases, requiring comparable data quality among datasets [25]. Findable, accessible, interoperable, and reusable (FAIR) standard guidelines have been developed for the identification, citation, and reporting of data and metadata in databases and repositories [26,27]. A growing body of work that aligns genomic data portals with these good data sharing practices is part of an ongoing process [28,29]. Once retrieved and pre-processed, omics data integration can be performed using a plethora of statistical methods, from correlation to multivariate analysis, as well as AI methods such as clustering or regression, which can approach missing data, collinearity and dimensionality, as issues intrinsically related to a multi-layer data analysis [30].

Finally, there is the aspect related to incrementally updating data to remain useful for re-analysis purposes. As shown in Table 1 and Figure 1, transcriptomics is experiencing an increasing trend in growing data production, and while one research group may stop data retrieval at a certain moment to begin their analysis, elsewhere in the world others may already be uploading new, useful datasets or publishing new disease classifications based on novel transcriptomics evidence. Once published or stored in a database, these results cannot be updated based on new data or evidence until someone else initiates a new retrieval, integration, or analysis process. This represents a limiting factor for research progress that needs to be solved by automation and recurrent analysis as an inevitable process.

Overcoming this limitation could lead to improved personalized medicine, tailoring therapies for patients by using an incrementally updated omics-based classification. Incremental updating could increase the value of the information stored in databases, making live data constantly available and useful when generated, such as new transcriptome–phenotype correlations. An oncologist or a neurosurgeon with access to a hospital database containing clinically relevant and omics data for patients affected by a highly heterogeneous primary brain tumor, i.e., a glioma, could benefit from database incremental updating; for example, a database that relies on Artificial Intelligence (AI) methods of data retrieval, integration, and analysis, which cyclically performs incremental updating and re-analyzing phases of data stored. The doctors could receive an alert because of the revised World Health Organization (WHO) classification system, thanks to which prognosis and treatment options of gliomas can vary according to new classifications of tumor grades, as well as the integration of molecular profiles, including DNA methylation patterns, mutational landscapes, and gene expression signatures. This new classification was indeed published in 2021, overriding the previously used one from 2016 that was mainly based on tumor histological and immunohistochemical characteristics, and so affecting the diagnosis and staging of gliomas, as well as the consequent therapeutic approach [31]. As a result of this change in paradigm, different solutions have already been proposed to retrospectively re-classify gliomas whose data are present in The Cancer Genome Atlas (TCGA) repository [32,33], as well as multicentric [34] and single-institution [35] studies. This appears to be particularly important when defining the minimum information requirements to address patients correct diagnosis, prognosis, and therapeutic options based on new tumor classifications, and gene expression profiling represents a relevant aspect of these requirements.

**Figure 2:**
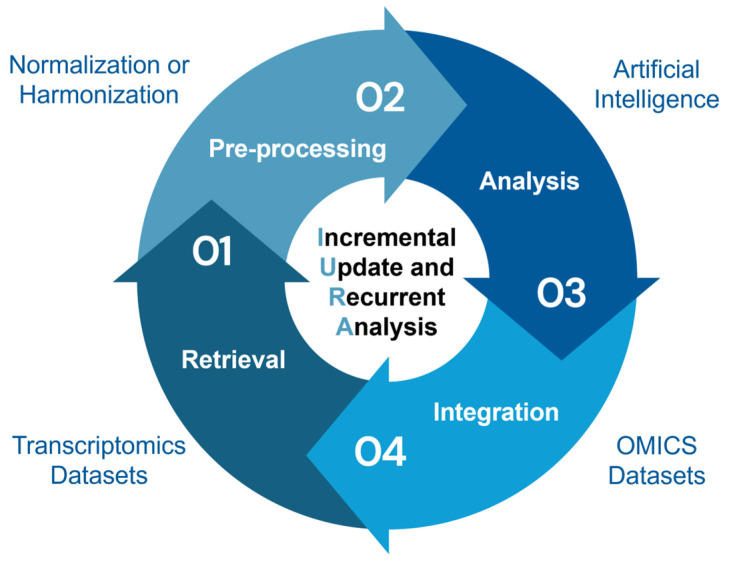
Proposed end-to-end data analysis pipeline. A semi-autonomous (or autonomous) data analysis of transcriptional profiles starting from a database retrieval phase of gene expression data, followed by pre-processing methods aiming to avoid platform bias. The data analysis phase relies on AI learning methods and continues with the integration of other omics data (including a new retrieval, pre-processing, and analysis phase of omics datasets). The incremental update and recurrent analysis (IURA) architecture could represent a further integration of the analysis pipeline. The figure was created by editing Circle Infographic provided by https://infograpia.com/ (accessed on 5 March 2026) and assembling them using PowerPoint by Microsoft.

Currently, the transcriptomic challenges related to data analysis are being addressed by computational systems biology approaches as bioinformatics increasingly relying on AI methods. As the growth and composition of transcriptomics databases switched in favor of next-generation RNA-seq techniques (Table 1), AI learning methods are also experiencing a flourishing period of development oriented toward the analysis of these data types. This aspect makes the research of AI algorithms suitable for analyzing pre-RNA-seq data more difficult, especially if pre-RNA-seq needs to be integrated with next-generation RNA-seq data. Moreover, transcriptomics challenges in data analysis and integration could benefit from using automation and recurrent analysis; however, some of these aspects could be challenging even for an AI [3]. As described in the next paragraph, data analysis based on AI learning methods strictly depends on input data, and gene expression profiles could be affected by different types of bias (Table 2). Regarding bias concerning data heterogeneity, extensive work should be done by researchers when producing these data, using robust and balanced study designs while adhering to the standards sharing guidelines for reporting data and metadata. Moreover, prior to the retrieval phase, researchers should a priori define which studies can be compared and what characteristics are needed to retrieve comparable datasets. Once preliminary steps have been performed, a data retrieval of transcriptional profiles can start from databases, followed by pre-processing of data using the same or different technologies, analysis and integration with different omics utilizing AI learning methods. A further integration of this pipeline with the incremental updating and recurrent analysis (IURA) of gene expression data could be performed by a semi-autonomous (or autonomous) AI-based analysis system (Figure 2) which is able to choose which method to use for each phase.

The implementations mentioned above are needed to overcome human and computational limitations in retrieving, pre-processing, analyzing and integrating the vast amount of data produced in order to model, simulate, and predict biological processes and treatment response; identify biomarkers and hidden pattern; and infer transcriptome–phenotype correlations [36,37]. Prospectively, the IURA model could be applied to digital twins modeling of virtual organs or systems mirroring the natural course of either a disease or treatment [38,39,40], using both clinical and pre-clinical data, with the aim of reaching diagnostic and therapeutic outcomes in less time. Semi-autonomous (or autonomous) systems can simplify complex procedures prior to and following data analysis, while at the same time amplify human capabilities in exploring biological complexity.

To the best of our knowledge, this is the first review to examine the role of AI learning methods specifically in support of transcriptomics, addressing not only data analysis but also presenting a forward-thinking perspective on how semi-autonomous (or autonomous) AI systems could be applied in both research and clinical settings using an IURA model. In the next section, we will cover these aspects in more detail by providing a few cutting-edge examples of how transcriptomics analysis is currently being studied using AI methods, as well as a semi-autonomous (or autonomous) AI-based system perspective.

## 2. AI in Transcriptomics

Nowadays, AI is revolutionizing the transcriptome-based field in terms of integration and analysis [6,41,42,43,44,45]. Its capabilities rely on intrinsic predictive, inferential, reinforcement, and generative features, enabling both the prediction of potential outcomes (in predictive models) and the generation of new content (in generative models), as summarized in Table 3.

Machine learning (ML) represents a key subset of AI predictive models and has already catalyzed a revolutionary shift in transcriptome studies, transforming static gene lists derived from expression values into dynamic, predictive models of cellular and disease states [6,41,42,45], either for pre-RNA-seq or next-generation RNA-seq technologies. ML models excel at predicting transcriptome-based identification of biomarkers, disease classification and/or states, pathways, and regulatory networks using supervised (from labeled data) or unsupervised (from unlabeled data) learning methods [42]. In particular, supervised and unsupervised approaches provide powerful predictive and exploratory features, respectively [41]. For example, a recent application of supervised AI methods has been applied to pre-treatment bulk gene expression profiles from publicly available oncological clinical trial transcriptomic datasets to obtain patient response predictions to cancer therapy [46]. While feature selection strategies based on unsupervised ML methods allow the identification of informative features without predefined data labeling, minimizing data input bias and highlighting complex patterns when applied to single-cell transcriptomic studies [47].

Another type of ML method that has been applied to transcriptome-based data and used for gene regulatory networks and cell fate studies is the reinforcement learning (RL), which lies between predictive and exploratory methods and focuses on learning through its interactions. In particular, through the exploration of data using multiple reward steps with the environment (gene expression profiles), it represents a “learning from experience” AI method useful for recurrent analysis and decision-making tasks in the transcriptomic field [48].

By incorporating deep neural networks, ML methods have evolved into deep learning (DL) approaches, which can infer hidden patterns from unstructured input data, thereby improving inference power when applied to classification, networks orchestrating transcriptional programs, mRNA splicing regulation, and spatiotemporal gene expression dynamics [41,42,49]. At the transcriptomic level, AlphaGenome has been recently presented as an AI genomic analysis framework based on a DL model. It is able to predict gene expression from DNA sequences, from variant effects to regulatory elements, and can perform comprehensive genomic analysis [50]. While from a precision medicine perspective, deep neural networks with labeled data can be used for supervised learning tasks; for instance, a decentralized AI learning framework, called Swarm learning, has been used as a disease classifier for medical diagnoses of leukemia, tuberculosis, and coronavirus disease-19 (COVID-19) from patients’ blood transcriptomes [51].

As the complexity of these models increases, AI methods can enhance their predictive power by integrating multiple learning models simultaneously. As an application in transcriptomics and personalized medicine, an RL framework potentiated with DL has been used to identify early decision points controlling cell fate from different single-cell RNA-seq datasets in normal human blood cell development, cancer cells, mouse organ development, and cells responding to radiation damage [52].

The progress of AI learning models has culminated in the use of generative AI (GenAI) learning models, prompted by user inputs and developed using large language models (LLMs), which show their generative capabilities as a core property alongside the aforementioned predictive, inferential, and reinforcement capabilities. In transcriptome-based prediction, GenAI has recently enabled the creation of a flexible, transcriptome-based in silico experimentation framework capable of predicting gene expression profiling results modeling bulk or single-cell RNA-seq data [53]. This development could represent a valuable resource, serving as a generative gene expression profiling AI platform, as has already happened in proteomics with a protein structure modeling platform implemented with generative AI, the AlphaFold3 [54]. An interesting study has compared the supervised class prediction capabilities of generative pre-trained transformer (GPT) models in comparison with different supervised ML models on lung and kidney cancer gene expression datasets extracted from TCGA. The authors performed data extraction and pre-processing phases, used a small set of informative genes to fit within GPT’s context window (window limit of information processed per iteration), split the two lung and kidney datasets into training and test sets, and performed the comparison using different prompt techniques for GenAI. The results highlighted that, although GPT was not specifically designed for this task, it was still able to classify a reduced set of genes with performance not inferior to the supervised ML models. However, ML methods achieved better results with no computational cost of running the analysis, regardless of which prompt technique was used [55]. From a semi-autonomous (or autonomous) perspective of AI-driven analysis, these differences could also guide the choice between one method and another. Moreover, retrieval augmented generation (RAG) methods, used to dynamically retrieve and integrate information expanding the limited context window of GenAI, have been positively tested for regulatory compliance with drug information and clinical trial protocol evaluations [56].

**Figure 3:**
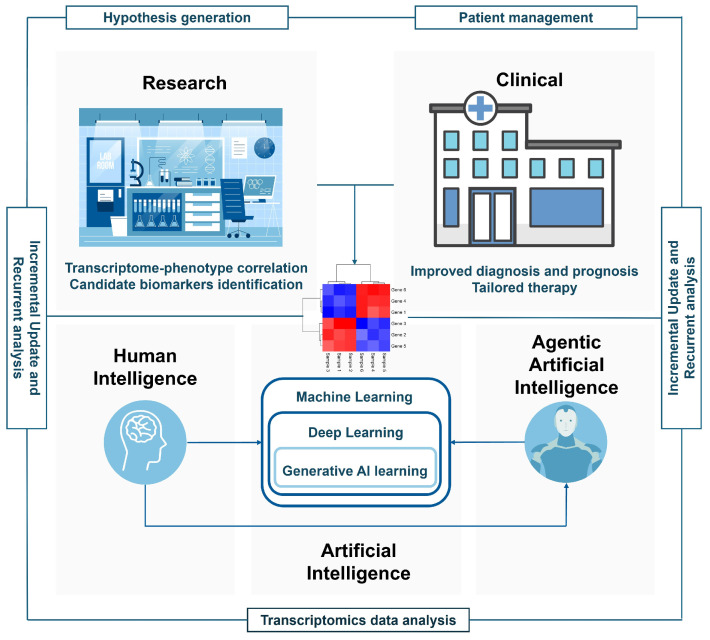
Proposed model for the use of agentic AI in personalized medicine. In the figure, agentic AI is depicted as a system able to follow a user-defined task on behalf of the user in an autonomous way, or to act proactively and independently (with no user intervention). Unlike traditional AI approaches that require human intervention at each step, agentic AI can autonomously carry on actions and make decisions to achieve specific objectives, including leveraging other AI methods, as a scientific agent or a co-scientist in a research team. The application of agentic AI to transcriptome-based analysis can be used for both basic research and clinical practice purposes, to perform automated and recurrent analysis (retrieval, pre-processing, integration, and analysis), incrementally update data, and generate new knowledge. The figure was created by editing (i) Artificial Intelligence and medical template infographics provided by https://infograpia.com/ (accessed on 1 December 2025), (ii) images of flat laboratory room (designed by Freepik) and hospital (designed by rawpixel.com/Freepik) from http://www.freepik.com (accessed on 19 January 2026), and (iii) creating the heatmap image using Kolde R (2025), pheatmap: Pretty Heatmaps, R package version 1.0.13, https://github.com/raivokolde/pheatmap (accessed on 19 January 2026), and assembling them using PowerPoint by Microsoft.

The methods described thus far still maintain the human-in-the-loop: humans decide which questions to ask; which actions to initiate; which methods to choose; which decisions to make; and, above all, retain responsibility for those decisions and resulting outcomes. However, a new revolution is emerging, i.e., the advent of agentic AI. As outlined in Figure 3, agentic AI can autonomously act following a user-defined task (on behalf of a human user) or defined objectives (in place of human user), taking actions and making decisions aimed at achieving these in a proactive manner, including the use of other bioinformatic resources and AI methods [57]. Theoretically, the difference between GenAI and agentic AI can be explained with what we could ask them. For example, we could ask GenAI a question (e.g., how can I perform a multi-omics data analysis?) and receive answers and examples (even functional programming codes); conversely, we could assign a task to an agentic AI (e.g., perform a multi-omics data analysis) and receive predictive models and functional interpretations, and the latter would pursue that end-to-end aim proactively using other AI methods as well. Moreover, an AI model able to bridge the gap between gene expression data and natural language discussion has been developed, enabling a chat-based exploration of single-cell RNA-seq datasets. This was the case for CellWhisperer, whose zero-shot prediction performance has been assessed for sample annotations of diseases, tissues, and organs rather than for cell-type retrieval only [58].

In general, when compared with AI learning methods so far described, agentic AI enable dynamic reasoning, workflow orchestration, verification, and adaptive refinement capabilities versus static or limited capabilities [59]. In this view, the application of agentic AI to transcriptomics studies can be beneficial at both basic research and clinical practice levels, accelerating discoveries and improving patient management. As described in Figure 2, an end-to-end data analysis pipeline performs retrieval, pre-processing of data from different transcriptomics studies, analysis using AI models, integration with other omics datasets, and allows data incremental updating and recurrent analysis, thereby generating new hypotheses and correlations (Figure 2 and Figure 3). Just to provide a few examples in research, these systems could be used to increase the number of samples screened over time, thereby enabling the discovery of new transcriptome–phenotype correlations or generate a list of new candidate biomarkers. Alternatively, in clinical practice, hospital databases could be updated with current evidence, allowing for modification of patient diagnosis, prognosis, and treatment in line with the latest official standards (e.g., the WHO gliomas classification) (Figure 3).

As a form of GenAI, agentic AI is developed using LLMs and comes with both advantages (e.g., zero-shot prompting enabling tasks to be set without providing examples) and disadvantages (e.g., hallucinations such as plausible but incorrect outputs as a probabilistic limitation of LLMs). Since agentic AI can act using other AI methods, it potentially possesses all the advantages of the previously described AI learning models, including predictive, inferential, reinforcement, and generative power, as well as the disadvantages, such as bias deriving from input data or reward hacking originating from RL. In particular, by taking advantage of learning from experience with minimal human supervision, a characteristic of RL methods, functional analysis and data processing exceeding human capacity could be approached by scientific agents [48], but this would incur reward misspecifications [60]. Despite the disadvantages that must be carefully considered and minimized, the aforementioned features, together with the capacity to act autonomously, could make agentic AI an enhanced instrument in the service of personalized medicine. AI agents can carry out complex tasks, simultaneously process huge amounts of data, integrate them from different sources, and update them in a time that is considerably shorter than that required by scientists. All of this can be achieved with the same internet connection and computing power requirements as those needed by a researcher using AI methods.

Examples of scalable frameworks for large multi-omics datasets that offer the opportunity to be implemented in an agentic view have been recently described for multi-omics data analysis in precision oncology. The first example was allowed to use RAG with biological networks analysis through graph neural networks (GNNs) methods. Starting from heterogeneous networks representing interactions, regulatory relationships, or signaling pathways, the framework integrated multi-omics patient-derived information, predicted models, and offered a functional interpretation through dynamically retrieved knowledge derived from the literature. This RAG-GNN framework was described to identify candidate therapeutic targets in the presence of specific gene mutations by allowing an integration of multi-omics patient-derived data, including gene expression profiles [61]. Another example was an agentic RAG-driven multi-omics analysis framework that allowed data collection and pre-processing, used an agentic system to dynamically retrieve and synthesize knowledge, and performed multi-omics data integration (from genomic, proteomic, transcriptomic, and metabolomic resources) and prediction. Through its use, a signaling pathway was investigated in colorectal cancer, taking advantage of an agentic RAG-based knowledge extraction system able to retrieve, synthesize knowledge, and generate hypothesis using LLMs, and of a GNNs-based data integration to integrate data and perform predictive models [62].

Although the short-term costs of implementing agentic AI in healthcare are substantial (including development, computing, and management expenses), these systems may ultimately become cost-saving resources by reducing long-term operational expenses and improving the return on investment [63]. In particular, the application of AI methods in healthcare has demonstrated substantial cost and time savings in both diagnostic and therapeutic settings compared with conventional approaches. These benefits include shorter waiting times for accurate diagnosis and treatment, as well as a more efficient patient management [64]. Moreover, the application of agentic AI in clinical practice could also reduce health inequality. While the great challenges for low-income countries are represented by data production and development of agentic AI systems, semi-autonomous (or autonomous) AI-based analysis systems, using incremental update and recurrent analysis, could facilitate the work of researchers and clinics all over the world. In fact, once data have been publicly shared and agentic AI developed, semi-autonomous (or autonomous) AI-based analysis systems using incremental update and recurrent analysis could achieve diagnostic and therapeutic objectives in a reduced timeframe. For example, transcriptomic diagnostic, prognostic, or therapeutic biomarkers selected using agentic AI could be easily detected via point-of-care assays transported to remote locations, with a decreased need for large and specialized diagnostics facilities [16]. Given these premises, agentic AI could provide a significant advantage when integrated into precision medicine.

While several scientists are still skeptical about the level of autonomy achieved by agentic AI [65], its integration into transcriptome-based research is already a plausible scenario, either as frameworks for autonomous multi-omics agentic AI-driven transcriptomic analysis have been developed with minimal input user requirements, or as fully automated end-to-end bioinformatics analysis agents based on LLMs [66,67]. This is the case of AutoBA, which stands for Automated Bioinformatics Analysis, and requires a data path, description, and task as its user input, which was designed as an autonomous AI agent for fully automated multi-omics analyses able to generate codes for sub-tasks thanks to LLMs [66]. Alternatively, a MEDEA agent can generate and execute a multi-step analysis plan when given a user input task, as well as verify either the literature context or plan integrity through different modules. This multi-omics analysis agent was also used to predict the personalized response to immunotherapy of patients affected by bladder urothelial carcinoma, using their tumor transcriptomic profiles [67]. These examples demonstrate the feasibility of omics data analysis and integration using AI and agentic AI.

Another example of agentic AI is represented by Agentomics-ML, a fully autonomous agent-based system able to produce ML models for class prediction tasks, requiring only dataset files (and optional description) as a user input and autonomously enabling the splitting of test and training data, as well as the inference on external datasets. Since file output steps are validated for the correct syntax and format, Agentomics-ML is forced to retry each failed steps and, after each iteration, a system of feedback guides the decision-making process for further steps [68]. With the aim to automate gene expression analysis, GenoTEX was implemented by a team of bioinformaticians as a standard pipeline for the automated analysis of gene expression data through the phases of data selection, pre-processing, analysis, and evaluation of the obtained results. Moreover, GenoTEX was used as a benchmark to assess a team of multi-LLM-based agents, called GenoAgent, for an end-to-end gene expression data analysis; as a result, this can highlight the limitations in the self-correction mechanisms and clinical feature extraction, as well as the importance of the data selection phase, since errors in this step can propagate through the pipeline impacting the overall performance, obtaining the highest score in pre-processing tasks [69]. GenoTEX was also used as a benchmark to assess another multi-LLM-based agent called Genomic data analysis through LLM-based Multi-Agent System (GenoMAS). As a multi-agent framework, GenoMAS enabled end-to-end code generation, revision, and validation for complex genomic analysis tasks through automated data selection, pre-processing, and analysis. Assessed with the GenoTEX benchmark, GenoMAS achieved a high accuracy in gene identification and obtained a higher performance in data pre-processing compared to GenoAgent [70].

In recent years, several LLMs-based AI agents have been developed or used for single-cell transcriptomics analysis, such as CellAgent, Agentic Triage of Regulated single-cell data Ingestion and Analysis (CellAtria), Embedding-Linked Interactive Single-cell Agent (ELISA), and DeepSeq. CellAgent is a multi-agent framework for single-cell RNA-seq analysis that takes advantage of a self-iterative optimization mechanism. When tested with different methods, it obtained the highest performance in cell type annotation [71]. CellAtria is an AI agent able to coordinate an automated end-to-end single-cell RNA-seq analysis pipeline called CellExpress in less than 10 min of running time [72]. ELISA can be queried using either a gene signature and/or natural language to generate candidate hypotheses through LLM reasoning. In comparison with CellWhisperer, it surpassed its performance in cell type retrieval [73]. Lastly, the augmented agentic GenAI of DeepSeq can be used for cell type annotation and prediction via web searching [74].

A benchmark was also developed as an open-source evaluation system for agent-based single-cell multi-omics analysis. It integrated different LLMs deployed within agent frameworks, including 50 analysis tasks and 18 evaluation quantitative metrics. Their implementation for single-cell multi-omics analysis confirmed that the use of multi-agent frameworks increased efficiency over a single-agent approach through task division, and that real-time error correction, adaptive strategy refinement, and RAG contribution had a major role in performance enhancement [75]. However, while the reported examples of agentic AI are highly heterogenous in scope (multi-omics, gene expression or single-cell RNA-seq data analysis), architecture (single-agent, multi-agent, or hybrid LLMs-based), and levels of task performance, flexibility, and automation, they confirm that the application of agentic AI to the transcriptomic field is in an early emerging stage for development and adoption, yet it could be essential to overcome human limitations. In Table 4, we summarized the advantages of agentic AI use in automated analysis of gene expression over a human-driven approach, where human-driven workflows are manual or semi-automated, pipeline-dependent, not systematic, and time-consuming, while the agentic-driven workflows promise to be automated, time-saving, and systematic, allowing flexible exploration of biological complexity and assisting in the generation of literature reviews and hypotheses.

In the future, agentic AI could autonomously design and execute in silico gene expression experiments, generate novel hypotheses, and integrate multi-omics data. They will contribute to accelerating discoveries at a speed with which the human brain cannot compete. Such advancements will help drive new discoveries and improve precision medicine, but they could also compromise research quality if not validated by scientists, as agentic AI is susceptible to input data bias, hallucinations, and reward hacking, which could affect the robustness of results and the decision-making process. Data selection, data generation, and decision-making represent sensitive phases that require dedicated mitigation strategies (such as data audits, self-correction mechanisms, evaluation metrics, and constraint-based design) and human supervision for both input data and the decision process, as well as human involvement in the validation of results.

These are only some of the ethical concerns surrounding agentic AI. In fact, transparency and explainability in AI-driven decisions, as well as data privacy, especially when handling sensitive genomic or clinical information, are other critical issues [41,51]. In this perspective, the Swarm learning’s decentralized framework, which uses a blockchain-based peer-to-peer sharing of model parameters but not confidential information, represents a strategy that could maintain confidentiality, security, and ethical standards of patients’ data [51] and could also be locally implemented with the IURA model. Finally, accountability for decisions made is paramount, and decisions based on flawed outcomes could negatively impact on public health and society as well as those depending on behavioral rules [76]. Decisions cannot be fully delegated to AI, and scientists remain accountable for them. To entirely realize the benefits of agentic AI in a field relevant to public health, it is crucial to establish robust ethical guidelines promoting interdisciplinary collaboration between professionals of different fields. The guidelines so far proposed [65,76] have primarily suggested to constrain agentic AI within certain limits, track its actions, and validate the outcomes through human intervention, thus prioritizing risk control over autonomous capabilities [76]. These aspects are also covered by the European AI Act [Regulation (EU) 2024/1689], although agentic AI is not yet explicitly defined in the document [77]. In fact, the definitions of an AI system in article 3(1) and of a general-purpose AI model in article 3(63) are sufficient to cover AI agents (as clarified in https://ai-act-service-desk.ec.europa.eu/en/faq, accessed on 17 March 2026). Moreover, agentic AI, when applied to the public health field, falls within the definition of a high-risk AI system. As a result, it is subjected to the same obligations about risk management, data quality, technical robustness, transparency, and human oversight (Chapter III AI Act).

## 3. Conclusions

AI methods are helping scientists to approach transcriptomics data retrieval, integration, and analysis, while managing data that are continuously growing. Thanks to their intrinsic features, these methods can enable retrieval, integration, and analysis of data stored in a recursive way, while incremental updating and recursive analysis can overcome human limitations. To address transcriptomics challenges, accelerate discoveries, and bring personalized medicine a step forward, AI agents could emerge as powerful allies for scientists in the models proposed. Personalized medicine could benefit from the use of AI agents, overcoming human limitations related to the analysis of transcriptomic profiles and thus, accelerating new discoveries and correlations. The responsible integration of agentic AI into transcriptomics studies holds the promise to accelerate scientific breakthroughs and prioritize risk control over its autonomous capabilities to ensure that these advances are beneficial for both science and society.

## Figures and Tables

**Table 1:** A list of public transcriptomics repositories.

Databases	Microarray, SAGE, EST, ISH	Bulk RNA-Seq	Single-Cell/Nucleus RNA-Seq	Spatial RNA-Seq
**Comprehensive**	GEO, ArrayExpress, Expression Atlas, Bgee.	GEO, ArrayExpress, Expression Atlas, Bgee, SRA.	GEO, ArrayExpress, Single-Cell Expression Atlas, Bgee, SPASCER, Tabula Sapiens, Tabula Muris, SRA, PanglaoDB, HCL.	GEO, ArrayExpress, SRA, SPASCER.
**Tumor-Specific**	CGAP Human SAGE, TCGA, GDC Data Portal, CellMiner CDB, CGGA, IVY GAP.	GDC Data Portal, TCGA, CellMiner CDB, CGGA, IVY GAP, OpenPBTA, OpenPedCan, Gabriella Miller Kids First Data.	GDC Data Portal, CGGA, SORC Database, SCI Myeloid Cell Atlas, Gabriella Miller Kids First Data, ScPCA.	HTAN Spatial Transcriptomics, SORC Database, CROST, STOmics DB.
**Organ, Tissue or Cell-Specific**	Allen Human Brain Atlas, BrainSpan Atlas, HBT, NIH Blueprint NHP Atlas, MGI GXD, BrainTx, SAGE Genie.	BICCN, BrainSpan Atlas, MGI GXD, GTEx.	BICCN, Spatial Mouse Atlas, SCI Myeloid Cell Atlas, Single-Cell Atlas of the Mouse Embryonic Heart, MGI GXD, STAB2.	BICCN, Spatial Mouse Atlas, MGI GXD.
**Disease or Treatment-Specific**	iLINCS, dbGaP, CellMiner CDB, IVY GAP, TARGET.	iLINCS, ALS Knowledge Portal, NEUROMINE Data Portal, dbGaP, AD Knowledge Portal, CellMiner CDB, IVY GAP, Aging, Dementia, and TBI Study, TARGET.	ALS Knowledge Portal, CMDGA, dbGaP, AD Knowledge Portal, ssREAD.	dbGaP, ssREAD.

Abbreviations: Serial Analysis of Gene Expression (SAGE); Expressed Sequence Tag (EST); In Situ Hybridization (ISH); Gene Expression Omnibus (GEO); Database for Gene Expression Evolution (Bgee); Sequence Read Archive (SRA); SPAtial transcriptomics annotation at Single-CEll Resolution (SPASCER); Panglao Database (PanglaoDB); Cancer Genome Anatomy Project (CGAP) uses SAGE; Human Cell Landscape (HCL); Genomic Data Commons (GDC) Data Portal; The Cancer Genome Atlas (TCGA); CellMiner Cross-DataBase (CDB); Chinese Glioma Genome Atlas (CGGA); IVY Glioblastoma Atlas Project (GAP); Open Pediatric Brain Tumor Atlas (OpenPBTA); Open Pediatric Cancer (OpenPedCan) Project; Single-Cell Pediatric Cancer Atlas (ScPCA); The Spinal Cord Injury (SCI) Myeloid Cell Atlas; Spatio-Temporal Cell Atlas of Brain (STAB2); Human Tumor Atlas Network (HTAN); Spatial Omics Resource of Cancer (SORC) Database; Comprehensive Repository of Spatial Transcriptomics (CROST); Spatial Transcript Omics DataBase (STOmics DB); Human Brain Transcriptome (HBT); National Institutes of Health (NIH) Blueprint Non-Human Primate (NHP) Atlas; Mouse Genome Informatics Gene Expression Database (MGI GXD); Adult Genotype–Tissue Expression (GTEx) Project; Brain Transcriptome (BrainTx) Database; Brain Initiative Cell Census Network (BICCN); Integrative Library of Integrated Network-Based Cellular Signatures (iLINCS); Database of Genotypes and Phenotypes (dbGaP); Alzheimer’s Disease (AD) Knowledge Portal; Aging, Dementia and Traumatic Brain Injury (TBI) Study; Common Metabolic Diseases Genome Atlas (CMDGA); Therapeutically Applicable Research to Generate Effective Treatments (TARGET); Single-Cell and Spatial RNA-Seq Database for Alzheimer’s Disease (ssREAD).

**Table 2:** A list of heterogeneity sources in transcriptomics data and related issues.

Data Heterogeneity Source	Issue
Technological platform	Platform bias
Batch effects	Technical variability
Record of metadata	Missing or incomplete information
Poor sample quality	Increased noise or degraded signal
Experimental phases not automated	Operator-dependent variability
Number of replicates (samples vs controls)	Affected statistical power and variance estimation
Biological material	Cellular composition differences
Sex-specific differences	Sex-dependent variability

**Table 3:** AI learning methods and their main tasks applied to transcriptomic analysis.

AI learning Method	Task
Machine learning	Classification, Regression, Clustering, Feature selection, Dimensionality reduction
Reinforcement learning	Sequential decision-making, Strategy optimization, Recurrent analysis
Deep learning	Pattern recognition, Prediction
Generative AI	Data generation, Data augmentation.

**Table 4:** Agentic AI advantages in transcriptional analysis.

Transcriptomic Challenges	Human-Driven Approach	Agentic AI Approach
**Data and metadata retrieval**	Manual search and download, or bioinformatic pipelines use	Semi-automated querying, downloading, and metadata parsing
**Data heterogeneity**	Ad hoc detection and correction, time-consuming	AI-assisted detection, systematic and adaptive correction strategies
**Data analysis**	Fixed sequential pipelines, manual parameter tuning	Iterative exploration of parameters and methods within flexible frameworks
**Integration with other omics**	Manual, complex, and time-consuming	Semi-automated multi-omics integration
**Biological interpretation and inference**	Manual literature review and hypothesis generation	AI-assisted literature retrieval, synthesis, and hypothesis generation
**Incremental update and recurrent analysis**	Not systematic and not up to date	Configurable for periodic and systematic updates and reanalysis

## Data Availability

No new data were created or analyzed in this study. Data availability is not applicable to this article.

## Abbreviations

The following abbreviations are used in this manuscript:

AD	Alzheimer’s Disease
Agentic AI	Agentic Artificial Intelligence
AI	Artificial Intelligence
ALS Knowledge Portal	Amyotrophic Lateral Sclerosis Knowledge Portal
Bgee	Database for Gene Expression Evolution
BICCN	Brain Initiative Cell Census Network
BrainTx	Brain Transcriptome
CDB	CellMiner Cross-Database
CellAtria	Agentic Triage of Regulated Single-Cell Data Ingestion and Analysis
CGAP Human SAGE	Cancer Genome Anatomy Project Human Serial Analysis of Gene Expression
CGGA	Chinese Glioma Genome Atlas
CMDGA	Common Metabolic Diseases Genome Atlas
COVID-19	Coronavirus Disease-19
dbGaP	Database of Genotypes and Phenotypes
DL	Deep Learning
ELISA	Embedding-Linked Interactive Single-Cell Agent
EST	Expressed Sequence Tag
FAIR	Findable, Accessible, Interoperable, and Reusable
GAP	IVY Glioblastoma Atlas Project
GDC	Genomic Data Commons
GenAI	Generative Artificial Intelligence
GenoMAS	Genomic Data Analysis Through LLM-Based Multi-Agent System
GEO	Gene Expression Omnibus
GNN	Graph Neural Networks
GPT	Generative Pre-Trained Transformer
GTEx	Genotype–Tissue Expression
HBT	Human Brain Transcriptome
HCL	Human Cell Landscape
HTAN Spatial Transcriptomics	Human Tumor Atlas Network Spatial Transcriptomics
iLINCS	Integrative Library of Integrated Network-Based Cellular Signatures
ISH	In Situ Hybridization
IURA	Incremental Update and Recurrent Analysis
LLMs	Large Language Models
MGI GXD	Mouse Genome Informatics Gene Expression Database
MIAME	Minimum Information About a Microarray Experiment
MINSEQE	The Minimal Information about a high-throughput nucleotide Sequencing Experiment
ML	Machine Learning
NHP	Non-Human Primate
NIH	National Institutes of Health
OpenPBTA	Open Pediatric Brain Tumor Atlas
OpenPedCan	Open Pediatric Cancer Project
PanglaoDB	Panglao Database
Pre-RNA-seq	Pre-RNA Sequencing
RAG	Retrieval-Augmented Generation
RL	Reinforcement Learning
SAGE	Serial Analysis of Gene Expression
SCI	Spinal Cord Injury
ScPCA	Single-Cell Pediatric Cancer Atlas
SORC	Spatial Omics Resource of Cancer
SPASCER	Spatial Transcriptomics Annotation at Single-Cell Resolution
SRA	Sequence Read Archive
ssREAD	Single-Cell and Spatial RNA-Seq Database for Alzheimer’s Disease
STAB2	Spatio-Temporal Cell Atlas of Brain
STOmics DB	Spatial Transcript Omics Database
TARGET	Therapeutically Applicable Research to Generate Effective Treatments
TBI	Traumatic Brain Injury
TCGA	The Cancer Genome Atlas
WHO	World Health Organization
